# Diet and Medicinal Herbs as Adjunctive Approaches to Immune Homeostasis in Sjögren’s Disease

**DOI:** 10.3390/ijms27093762

**Published:** 2026-04-23

**Authors:** Xiaoyu Xu, Jie Yu, Yun Feng, Jing He, Xiang Lin

**Affiliations:** 1 School of Chinese Medicine, Li Ka Shing Faculty of Medicine, The University of Hong Kong, Hong Kong SAR 999077, China; 2 Department of Ophthalmology, Peking University First Hospital, Beijing 100034, China; 3 Beijing Key Laboratory for Rheumatism Mechanism and Immune Diagnosis, Department of Rheumatology & Immunology, Peking University People’s Hospital, Beijing 100044, China; 4 Department of Chinese Medicine, The University of Hong Kong-Shenzhen Hospital (HKU-SZH), Shenzhen 518053, China

**Keywords:** Sjögren’s disease, dietary, medicinal herbs, immune response

## Abstract

Sjögren’s disease (SjD) is a chronic autoimmune disorder characterized by progressive dysfunction of the exocrine glands, driven primarily by aberrant T- and B-cell activation. Current therapeutic strategies remain largely symptomatic and are frequently limited by off-target effects and long-term toxicity, underscoring an urgent need for safer, mechanism-based adjunctive approaches. In recent years, nutritional interventions and medicinal herbs have emerged as promising complementary strategies, owing to their capacity to modulate immune–metabolic pathways and restore immune homeostasis. Nutrients such as n-3 polyunsaturated fatty acids (PUFAs) and short-chain fatty acids (SCFAs) exert well-documented anti-inflammatory effects and influence immune cell differentiation via immunometabolic reprogramming. Concurrently, bioactive constituents derived from medicinal herbs offer multi-target regulation of inflammatory signaling and lymphocyte function. This review synthesizes current advances in the immunomodulatory roles of dietary components and edible herbs in the context of SjD, focusing on their mechanistic convergence on T-cell subsets, B-cell responses, and the gut–immune axis. By integrating traditional knowledge with contemporary immunological insights, this article aims to provide a conceptual framework for the rational integration of nutritional and herbal strategies into the clinical management of SjD.

## 1. Introduction

Sjögren’s disease (SjD) is an autoimmune disorder affecting the exocrine glands of the human body. Xerostomia and keratoconjunctivitis sicca are the hallmark manifestations of the disorder; nevertheless, these manifestations are not limited to the exocrine glands. Several patients also present with systemic manifestations of the disorder. For instance, patients with SjD often complain of arthralgia or fatigue [1]. From an immunological perspective, the disease is characterized by an abnormal attack on the salivary and lacrimal glands. This results in the infiltration of the affected tissues with large numbers of lymphocytes and, consequently, permanent tissue damage. This abnormal immune response causes localized tissue damage and results in the overall complications that define SjD [2].

Epidemiological studies show that SjD is much more common in women, particularly middle-aged to elderly women, with a female-to-male ratio of 9:1. Though researchers are still investigating this, most experts believe that this significant gender gap is associated with hormonal factors, as well as how immunity is regulated in each gender [3]. Furthermore, the frequency of SjD shows significant geographical variation. In Asia, the rate of SjD is approximately 40–60 per 100,000 people, while in Europe, it is slightly higher, at 60–80 per 100,000 people. Ultimately, the development of SjD involves a combination of genetics, the environment, and the endocrine system [4].

As a systemic condition, SjD is defined by the uncontrolled activation of T and B cells, where pro-inflammatory factors promote disease progression [5]. This process usually begins with an abnormal response to autoantigens. These endogenous antigens, such as Ro and La, are released and then prime dendritic cells and T cells to continue the autoimmune disease cycle. These cells then continuously attack glandular tissues, thus creating a condition of inflammation [6]. Meanwhile, hyperactive B cells produce autoantibodies, which, in turn, form complexes with self-antigens, thereby activating the complement cascade and causing more damage.

The management of SjD remains the same, i.e., attempting to manage the symptoms, as well as the immunological response. The current first-line management protocols include the use of glucocorticoids, as well as immunosuppressants, though these are far from ideal, as the risk of adverse effects, including osteoporosis, hepatorenal toxicity, and increased infection risk, is a major hindrance in the disease’s management. In addition, the drugs cannot produce consistent responses in patients, as they find it difficult to tolerate such a regimen [7]. Considering these disadvantages and the need for a more individualized approach, new alternatives with low toxicity must be urgently sought. This prompted the recent focus on dietary and herbal approaches to reset the immune system and improve patients’ quality of life with fewer side effects.

Recent research on immune regulation using herbs has shown that various herb-derived agents have significant anti-inflammatory and antioxidant properties. For example, medicinal herbs such as *Astragalus*, *Ganoderma lucidum*, *Lycium barbarum*, and *Allium sativum* have demonstrated immunomodulatory properties, which will be discussed in detail below.

This review aims to critically evaluate the use of dietary nutrients and edible medicinal herbs as supportive therapies for SjD, considering their role in the mechanistic modulation of the immune system. By bridging traditional dietary wisdom with contemporary immunometabolic insights, this review aims to establish a mechanistic foundation for the rational integration of nutritional and herbal strategies into evidence-based clinical management for SjD.

## 2. The Immunopathogenesis of SjD

Phenotypic mapping has highlighted the functional importance of CD4+ T cells and B cells in the pathology of SjD [8,9]. T cells play a key role in the secretion of pro-inflammatory cytokines and are responsible for the direct interaction with B cells through the formation of immune synapses. T cells, upon activation, infiltrate the exocrine glands, leading to the release of high concentrations of cytokines (e.g., TNF-a, IFN-γ, and IL-17). The release of these cytokines causes damage, leading to the destruction of gland functionality. The excessive activity of the Th1 and Th17 pathways, along with the presence of T follicular helper cells, signifies the pathological landscape of SjD [10,11,12]. This is reinforced by T-cell-mediated signals, which enhance B-cell dysfunction. Autoantibodies, including anti-SSA (Ro) and anti-SSB (La), are commonly elevated in the sera of SjD patients, serving as a disease marker and mediator of tissue damage through complement activation [13]. Thus, T cells and B cells actively modulate the systemic and glandular microenvironment, which increases glandular tissue damage by promoting lymphocyte chemotaxis and the inflammatory cascade [14]. For example, the production of effector molecules, including IL-6, by cells such as dendritic cells, macrophages, and T cells, which may enhance B-cell differentiation, is a key mediator of disease progression [15]. Upon initiation of the inflammatory cascade, the affected tissues become dysfunctional, leading to a state of chronic inflammation [16].

Emerging evidence also highlights the critical involvement of myeloid cell subsets—including monocytes, macrophages, and plasmacytoid dendritic cells (pDCs)—in SjD pathogenesis. Single-cell transcriptomics has revealed a novel monocyte subset with high VNN2 and S100A12 expression that is expanded in SjD, alongside upregulation of *TNFSF10* (TRAIL) across most monocyte subsets, suggesting a pathogenic role [17]. A consistent M1/M2 macrophage imbalance has been further demonstrated: patients with primary SjD exhibit increased M1 and decreased M2 macrophages in blood and salivary glands, with elevated M1-related cytokines (IL-6, IL-23, TNF-α) and reduced IL-10; M1 proportion correlates positively with IgG and rheumatoid factor, linking M1 polarization to disease activity [18,19]. Furthermore, pDCs drive early disease in the NOD mouse model via type I interferon signaling; pDC depletion reduces glandular inflammation, lymphocyte infiltration, and pro-inflammatory factors while improving salivary flow, although autoantibody levels remain unchanged [20]. Collectively, myeloid dysregulation—encompassing pathogenic monocyte subsets, M1-dominant polarization, and pDC-driven IFN responses—plays a central role in SjD pathogenesis and offers novel therapeutic targets.

## 3. Role of Dietary Supplementation in Regulating Immune Responses in SjD

**Figure 1:**
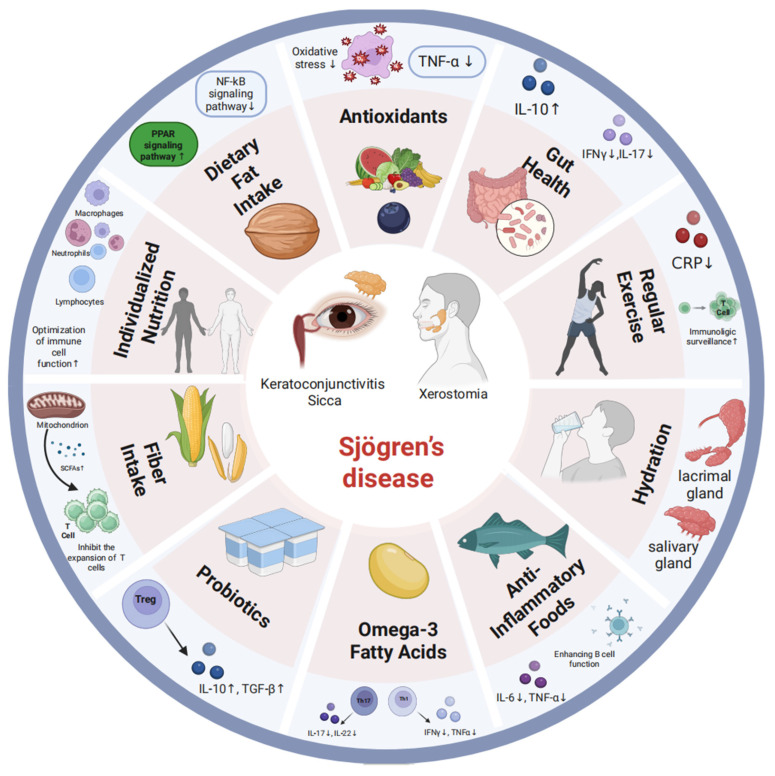
Dietary and lifestyle interventions for the management of Sjögren’s disease (SjD). This figure illustrates the multifaceted approach to managing SjD via dietary and lifestyle modifications. The diagram is organized into two main sections: dietary factors and their corresponding effects on immune cell functions and signaling pathways.

Dietary factors have profound effects on immune cell function and inflammatory signaling, offering a modulable entry point for intervention in SjD. Figure 1 provides a conceptual overview of this interplay, organizing dietary components on the left and their corresponding effects on immune cell subsets and signaling pathways on the right. Broadly, nutrients such as short-chain fatty acids (SCFAs), omega-3 polyunsaturated fatty acids (n-3 PUFAs), and dietary fibers exert immunoregulatory effects by shaping T-cell differentiation, modulating B-cell responses, and influencing the gut microbiota. In the following sections, we discuss in detail how specific dietary factors influence distinct lymphocyte populations—including B cells, CD4+ T-helper subsets, and regulatory T cells—and how these insights may inform dietary strategies in SjD.

### 3.1. Modulating B-Cell Response

Humoral autoimmunity is the hallmark of SjD, in which the aberrant activation of B cells is a significant immunological feature [11,21]. Plasma cells are antibody-secreting cells that differentiate from B cells following activation. By recognizing tissue-specific autoantigens, recent advances report that epithelial stromal interaction protein 1 is highly expressed in B cells in SjD and plays a regulatory role in the aberrant activation of B cells [22]. In SjD, plasma cells produce autoantibodies that target the body’s own tissues, particularly the salivary and lacrimal glands, thereby further activating the complement system, recruiting and stimulating immune cells such as macrophages and neutrophils, and amplifying the inflammatory response [23,24]. Despite the absence of clinical evidence of the impact of dietary factors on B-cell response in SjD, recent studies have indicated the multidimensional role of dietary factors in B cells, including immune regulation and metabolic processes. A large-scale isocaloric diet study in murine models revealed that high-carbohydrate diets—particularly those rich in glucose—significantly promote B-cell development and function [25]. While such enhancement may bolster general immunity, it carries the risk of exacerbating autoimmune inflammation, particularly in obese individuals. In contrast, high-fat diets have been shown to impair gut microbiota composition, leading to disrupted B-cell development and reduced IgA responses [26,27].

Meanwhile, (n-3) PUFAs, abundant in fish and nuts, can normalize B-cell function and downregulate pro-inflammatory signals [28]. Diets rich in SCFAs are also known to support the microbiome and regulate autoreactive T cell expansion by modifying B-cell co-stimulation and differentiation [29]. Finally, dietary fibers from whole grains and vegetables are essential nutrients for the growth of beneficial microbes such as Bifidobacteria, which can produce SCFAs such as butyrate and acetate from dietary fibers. These are essential in maintaining the integrity of the intestinal barrier and controlling inflammation. In contrast, Western diets rich in processed meats and sugars are known to induce dysbiosis and insulin resistance, which can contribute to the pathological activation of plasma cells [30].

In addition to effector subsets, another important immunomodulatory role of regulatory B cells (Bregs) is their ability to secrete anti-inflammatory cytokines that prevent or restrain an overactive immune response [31,32]. These cells may alleviate symptoms of SjD by mitigating chronic inflammation. Leucine-rich dietary intake has been observed to promote a novel subpopulation of Bregs, identified as Lars2-expressing B cells. These cells exhibit significant mitochondrial respiratory chain complex I inhibition, impaired NAD+ regeneration, reduced NAD+/NADH ratios, and diminished mitochondrial membrane potential [33]. These metabolic alterations activate NAD+-dependent enzymes, including SIRT1, which upregulates transforming growth factor beta-1 (TGF-β1) expression through the deacetylation of paired box 5, thereby contributing to a sustained and exacerbated inflammatory response that may drive disease progression in SjD [33] (Table 1). For patients with SjD, adopting a diet rich in (n-3) PUFAs and fibers to support SCFA production, while limiting saturated fats, may help attenuate B-cell hyperactivation and support immune homeostasis.

### 3.2. Modulating T-Cell Response

CD8+ T cells are crucial components of the cellular immune response. In SjD, these cells may aggravate tissue damage by recognizing self-antigens, triggering immune-mediated attacks, and releasing a spectrum of pro-inflammatory cytokines, ultimately resulting in tissue fibrosis and functional impairment [34]. High-fat diets (HFDs) promote the accumulation of CD8+ T cells in adipose tissue, contributing to chronic low-grade inflammation, especially in obese individuals, through interactions with macrophages [35]. Moreover, HFDs have been shown to elevate the levels of CD8+ T cells in the brains of aged rats, thereby exacerbating neuroinflammatory responses and impairing memory function [36]. Trans-oleic acid, a fatty acid abundant in human breast milk, enhances CD8+ T-cell functionality by inhibiting the GPR43 receptor and activating the cAMP-dependent protein kinase A-cyclic AMP response element-binding protein signaling pathway [37]. This finding may not only suggest a promising therapeutic target in cancer treatment but also a potential risk factor in autoimmune disorders such as SjD.

Memory T cells are a critical component of the adaptive immune response; however, in SjD, immune tolerance may become impaired, leading memory T cells to attack self-tissues and aggravate glandular infiltration [38]. Compiled evidence indicates that moderate caloric restriction, particularly when implemented without causing malnutrition, enhances memory T-cell functionality. This effect is likely due to the optimization of these cells’ metabolic state, thereby boosting the efficiency of their immune response [39]. Furthermore, a high-fiber diet supports the maintenance and functionality of memory T cells, primarily because SCFAs, e.g., butyric acid, enhance mitochondrial oxidative metabolism of effector T cells, thereby indirectly promoting systemic immune function [40].

T follicular helper (Tfh) cells are a subset of effector T cells and are essential in effector B-cell responses, aiding in the production of high-affinity antibodies. In patients with SjD, the frequency of circulating Tfh cells is increased and positively correlates with the disease severity [41]. Recent advances reveal that HFDs lead to an expansion of Tfh cells, exacerbating the inflammatory process by enhancing B-cell activation and antibody production. However, in contrast, marginal zone B (MZB) cells regulate Tfh cell differentiation and accumulation under HFDs by activating programmed death-ligand 1 expression. This regulatory role of MZB cells limits the excessive adaptive immune response [42]. A high-salt diet promotes Tfh cell differentiation through TET2-induced DNA demethylation, a mechanism that plays a crucial role in autoimmune diseases, such as systemic lupus erythematosus (SLE) [43].

Th1 cells are critical mediators of the adaptive immune response during SjD progression. They exhibit abnormal activation and dysregulated balance, which boosts the immune system to erroneously attack its own tissues [44], including anti-SSA/Ro and anti-SSB/La, and participate in glandular tissue damage [45]. This is achieved by the production of their predominant cytokines, including IFN-γ, along with other pro-inflammatory cytokines, thus amplifying inflammatory responses and resulting in systemic and glandular tissue destruction [8]. The effects of HFDs on Th1 cell response have multiple layers, but generally, they enhance Th1 differentiation and activation.

HFDs’ ability to enhance the secretion capacity of IFN-γ via Th1 cells was demonstrated in [46]. Detailed mechanistic studies have revealed that HFDs induce metabolic reprogramming that increases glycolysis and fatty acid oxidation, thereby providing the metabolic support required for Th1 cell activation and clonal expansion [47]. Additionally, HFDs may modulate Th1 cell function indirectly through their effects on the commensal flora. Murine models have shown that HFDs reduce beneficial flora while promoting pathogenic outgrowth; the resulting compromise of gastrointestinal barrier integrity allows bacterial products to enter systemic circulation, further exacerbating Th1-mediated inflammation [48].

Meanwhile, (n-3) PUFA-enriched diets have an immunosuppressive role in Th1 cell activity. Experimental research using C57BL/6 mice showed that diets enriched with fish oil resulted in a substantial reduction in Th1 cell frequency, even when Th2 polarization was attempted. A similar finding of increased Th2 cell levels suggests that (n-3) PUFAs may affect Th1/Th2 immunology [49]. Moreover, diets enriched with guar gum, a soluble fiber, resulted in substantial delays of the onset of experimental autoimmune encephalomyelitis, an animal model of human multiple sclerosis. Guar gum has been shown to affect Th1 cell activation, proliferation, and differentiation, as well as their migration ability [50].

Th17 cells represent another critical subset whose activity is profoundly influenced by dietary factors. Th17 cells are a subset of effector CD4+ T cells, with a pivotal role in host defense against extracellular pathogens, particularly at mucosal and epithelial barriers, by producing cytokine IL-17. However, when aberrantly activated, Th17 cells can produce excessive IL-17, leading to heightened inflammatory responses, infiltration of inflammatory cells, and subsequent tissue damage. Salivary epithelial cells highly express IL-17 receptors, conveying localized inflammation and tissue damage that manifest as glandular dysfunction and dryness symptoms [51].

Several dietary factors have been shown to promote Th17 differentiation. A high-salt diet facilitates Th17 differentiation and augments IL-17 production, an effect mediated, in part, via NLRP3 inflammasome activation and gut microbiota modification [52]. This effect is achieved, at least in part, through the activation of inflammasomes associated with NLRP3 [53,54], as well as altered gut microbiota species [55]. Similarly, high-fat diets and high-sugar diets have been associated with increased Th17 cell counts [43], accompanied by blood sugar levels [56]. Conversely, specific dietary elements impede Th17 differentiation and activation. (n-3) PUFAs, however, inhibit Th17 cell frequencies and are associated with enhanced regulatory T-cell (Treg) function [57]. These opposing effects highlight the importance of nutritional composition in maintaining Th17–Treg balance and immune homeostasis.

Treg cells are well-recognized to sustain immune tolerance by producing immunosuppressive cytokines [58]. Thus, HFDs, particularly those rich in trans and saturated fats, may impair Treg cell function and, thus, elevate the risk of chronic inflammation and autoimmune diseases [59]. Meanwhile, diets rich in antioxidants and polyphenolic compounds, such as those found in green tea, berries, and dark green vegetables, have been shown to promote the differentiation and function of Treg cells, thereby facilitating the suppression of overt immune responses [60,61]. Antioxidants, including vitamins C and E, neutralize free radicals, thus reducing oxidative stress-induced damage in response to an inflammatory microenvironment. This mechanism supports the stability and functionality of Treg cells [62]. Dietary polyphenolic compounds help to reduce oxidative stress, thus protecting Treg cells from trans-differentiation into effector phenotypes with preserved cell numbers and function [63]. Similarly, epigallocatechin gallate, a compound found in green tea, promotes Treg cell response [62]. Furthermore, dietary anthocyanins and flavonoids, rich in berries, are also beneficial to health with enhanced Treg cell function [64].

Nutrients in these foods also work together to impact the metabolic health of Treg cells and alter their immunoregulatory capacity. Trans and saturated fats from HFDs and a high-salt diet also impede Treg cell function during chronic inflammation and autoimmune diseases, while (n-3) polyunsaturated fatty acids and SCFAs facilitate Treg cell differentiation indirectly.

Myeloid-derived suppressor cells (MDSCs), similar to Treg cells, are a heterogeneous population of myeloid cells with strong immunosuppressive capacity [65]. Recent studies have shown that tryptophan metabolites (such as indole-3-propionic acid (IPA)) can significantly promote the differentiation and function of polymorphonuclear myeloid-derived suppressor cells (PMN-MDSCs), primarily through the activation of the aryl hydrocarbon receptor (AhR) [66]. The AhR is a cytoplasmic receptor that can respond to various exogenous and endogenous compounds and is widely present in the gut microbiota, host metabolism, and environment [67,68,69]. In addition to IPA, the natural ligands of AhR also include various compounds derived from foods and herbs, such as resveratrol (a polyphenolic compound found in grape skins and certain berries), curcumin (derived from turmeric), and certain fungal metabolites [70]. These natural ligands regulate the activation and function of immune cells by binding to the AhR, thereby affecting inflammatory responses and immune balance. In the pathogenesis of SjD, activation of the AhR may alleviate immune-mediated tissue damage by enhancing the immunosuppressive function of PMN-MDSCs, providing a new potential target for the immunomodulatory treatment of SjD.

**Table 1:** Dietary recommendations for SjD.

Food Type	Specific Recommendations	Mechanisms of Action	References
Short-chain fatty acids (SCFAs)	Increase intake of dietary fiber, such as whole grains, legumes, and vegetables	SCFAs regulate the gut microbiota, enhance gut barrier function, inhibit the expansion of autoreactive T cells, and reduce inflammation.	[29]
Leucine	Consume an appropriate amount of leucine-rich foods, such as lean meats, eggs, and legumes	Leucine promotes the generation of Bregs, which secrete anti-inflammatory cytokines, reducing chronic inflammation.	[33]
Low-salt diet	Reduce salt intake and avoid processed and high-salt foods	High-salt diets can promote the differentiation of Th17 cells, increase the production of pro-inflammatory cytokines, and exacerbate inflammatory responses.	[43]
High-fiber diet	Increase intake of dietary fiber, such as whole grains, legumes, and vegetables	Dietary fiber ferments to produce SCFAs, maintaining gut microbiota balance, enhancing the function of memory T cells, and supporting systemic immune function.	[50]
Omega-3	Increase intake of fish, flaxseeds, and walnuts	Omega-3 fatty acids inhibit the overactivation of Th1 and Th17 cells, reduce the production of pro-inflammatory cytokines, and maintain immune tolerance.	[57]
Antioxidants	Increase intake of fresh fruits (such as blueberries and strawberries) and vegetables (such as spinach and broccoli)	Antioxidants (such as vitamins C, E, and polyphenolic compounds) neutralize free radicals, reduce oxidative stress, protect immune cells, and maintain immune tolerance.	[62]

## 4. Immunomodulatory Effects of Edible Herbs

In addition to dietary nutrients, edible medicinal herbs, owing to their diverse bioactive components such as polysaccharides [71], flavonoids [72], and polyphenols, have been extensively studied and utilized in immune regulation. The immunomodulatory effects of herbs have been recently demonstrated to be a key mechanism in treating SjD [73,74]. These natural bioactive compounds are pivotal in the prevention and treatment of various inflammatory diseases, which is beneficial in restoring immune tolerance [75,76,77]. These herbs have usually been reported to exhibit multi-target actions and multi-component regulation, improving patient outcomes. For example, active constituents in herbs, such as astragaloside IV from *Astragalus*, polysaccharides from *Ganoderma lucidum*, and polysaccharides from *Lycium barbarum*, all showed pronounced anti-inflammatory and immunomodulatory effects, albeit through distinct molecular pathways [78,79,80,81] (Table 2).

Clinical practice using medicinal herbs suggested that symptoms of dryness associated with SjD were alleviated, including xerostomia and keratoconjunctivitis sicca, as well as mitigating fatigue, thereby enhancing patients’ overall quality of life. Extracts from *Astragalus* have shown significant efficacy in enhancing SG function and alleviating dry mouth symptoms [82]. Moreover, the combination of peony and hydroxychloroquine can enhance immune regulatory effects while mitigating drug toxicity [83]. These mechanisms offer a theoretical foundation for the application of herbal therapies in the treatment of SjD and provide new options for the comprehensive management of SjD. Herein, we first review the available clinical trials using traditional Chinese medicines, followed by the introduction of several edible medicinal herbs that may benefit patients.

### 4.1. Clinical Evidence of Chinese Medicine in SjD

Over the past decade, a growing number of randomized controlled trials (RCTs) have evaluated the clinical efficacy of TCM in SjD, with several promising signals emerging. A multi-center, double-blind, placebo-controlled trial assigned 320 pSS patients to receive total glucosides of peony (TGP) 600 mg three times daily or placebo for 24 weeks; the TGP group showed significantly greater improvement in ESSPRI (*p* < 0.001), dry eye VAS, fatigue VAS, PGA, Schirmer‘s test and ESR, with only mild diarrhea (4.8%) as the main adverse event [84]. A smaller double-blind trial added JieDuTongLuoShengJin granules (consisting of *Paeoniae*, *Zedoariae*, *Angelica* and *Astragalus*) to hydroxychloroquine (HCQ) in 40 low-activity pSS patients; the combination significantly improved ESSPRI and PGA compared to HCQ alone, and the treatment group also showed significant within-group improvements in unstimulated salivary flow and IgG, though between-group differences did not reach significance for these objective measures [85]. Similarly, in a pilot RCT, Wu et al. also conducted a comparison between HCQ alone and in combination with an herbal formula (containing *Ophiopogonis*, *Paeoniae*, *Polygonati*, *Salviae Miltiorrhizae*, *Artemisiae Annuae*, *Notoginseng* and *Puerariae*) in 68 pSS patients over 3 months; the combination led to significantly greater reductions in IgG, ESR and osteopontin (OPN) levels, as well as improvements in several health domains (SF 36 scores, *p* < 0.05) [86]. Meta-analyses further support the overall superiority of TCM over conventional Western medicine. Liu et al. [87] (2016) pooled 31 RCTs (2137 patients) and found a significantly higher effective rate for TCM monotherapy (87.18%) compared to Western medicine alone (65.63%), with an odds ratio of 3.74 (95% CI: 2.99–4.69). A more recent network meta-analysis (2024) including 66 RCTs (5052 patients) concluded that TCM combined with conventional Western medicine is significantly more effective than first-line medication alone across multiple outcome indicators.

Despite these encouraging findings, several methodological limitations must be acknowledged. First, sample sizes are relatively small (n = 40; Wu et al. n = 68), and most trials have short follow-up periods (6–24 weeks), insufficient to assess long-term efficacy, durability of response, or delayed adverse events [85]. Second, TCM formulas are complex, multi-herb mixtures with wide inter-study heterogeneity; the optimal composition, dosing regimen, and pharmaceutical standardization remain to be clearly defined, which may hinder further replication, direct comparison, and meta-analytic synthesis. Third, pharmacokinetic data, active constituent profiling, and quality control information for TCM products are largely lacking. Future research should prioritize large-scale, multi-center, double-blind, placebo-controlled trials with longer observation periods (≥48 weeks) to confirm sustained efficacy and safety. Outcome assessment should adhere to internationally validated instruments (ESSDAI/ESSPRI) and include sensitive, objective biomarkers of glandular function, such as salivary scintigraphy or novel imaging modalities. Mechanistic studies exploring the immunomodulatory pathways of specific TCM compounds are needed to identify active constituents and enable targeted development. Finally, rigorous pharmaceutical quality control and standardization of TCM formulas—through modern analytical methods—are essential prerequisites for reproducible, evidence-based integration of TCM into SjD management guidelines.

### 4.2. Astragalus

Astragalus is a widely utilized medicinal herb renowned for its ability to modulate immune responses and, importantly, is the most frequently prescribed medicinal herb in clinical management [88,89]. Research has demonstrated that Astragalus plays a significant role in immunomodulation. Astragalus polysaccharide (APS), one of its major active components, enhances immune function by activating various immune cells, including macrophages, natural killer (NK) cells, and T lymphocytes [90,91]. Mechanistic studies have revealed that APS improves CD8+ T-cell function by regulating the STAT3/Gal-3/LAG3 signaling pathway, thereby exerting immunomodulatory effects in inflammation-related diseases. In addition, Astragalus exhibits potent anti-inflammatory activity. Total flavonoids extracted from Astragalus have been shown to significantly reduce the expression of pro-inflammatory cytokines (such as TNF-α, IL-6, and IL-1β) in lipopolysaccharide (LPS)-stimulated RAW 264.7 macrophages. These effects are mediated by inhibiting the activation of the NF-κB and MAPK signaling pathways [92]. Specifically, these compounds block the nuclear translocation of the p65 protein and downregulate the phosphorylation levels of ERK, JNK, and p38 MAPK, thereby alleviating inflammatory responses. Clinically, the application of Astragalus has also demonstrated promising therapeutic potential. A clinical trial involving patients with Sjögren’s disease (SjD) showed that Astragalus effectively alleviated dry mouth symptoms, suggesting its value in modulating immune dysregulation and reducing glandular inflammation [93].

Mechanistic studies demonstrated that the improvement might be linked to the astragaloside-induced rise in intracellular calcium ion levels and the activation of protein kinase C (PKC) alongside tyrosine kinases, including Lck and Fyn [94,95]. Once activated, these kinases promote the initiation of critical nuclear transcription factors. Consequently, this process enhances cytokine expression, including IL-2 and IFN-γ [96,97]. *Astragalus polysaccharides* (APSs) can modulate T-cell proliferation during activation by enhancing the signaling pathways of T-cell receptors (TCRs) and co-stimulatory molecules, including CD28 [98]. Meanwhile, APS also amplifies TGF-β signaling, consequently boosting the expression of the Foxp3 gene, a crucial transcription factor for Treg cell development [99]. Stable Foxp3 expression allows Treg cells to effectively suppress the activity of other immune cells. This regulation plays a vital role in maintaining immune tolerance and preventing autoimmune responses, thus ensuring the immune system operates correctly without targeting the body’s own tissues [100]. APS facilitates the proliferation of B cells and their differentiation into Breg [101] and plasma cells [102]. This process increases immune regulation and antibody production, thereby strengthening the body’s immune response against pathogens and maintaining immune homeostasis.

### 4.3. Dendrobium

*Dendrobium* is a medicinal plant known for its diverse pharmacological activities, and its primary chemical constituents include polysaccharides, gigantol, dendrobin, moupinamide, and isoliquiritin [82]. Extensive investigations have shown the various pharmacological properties of this medicinal plant, including immunomodulation [103], blood glucose regulation [104], and anti-cataract effects [105]. For instance, polysaccharides derived from *Dendrobium officinale* can significantly decrease levels of inflammatory cytokines in the retina and systemically in rats with diabetic retinopathy by inhibiting retinal VEGF expression [106]. These effects may help alleviate dry eye symptoms and the systemic inflammatory state observed in patients with SjD. In a mouse model of experimental SjD (ESS), Dendrobium polysaccharides enhanced SG function by enhancing their cholinergic response and promoted Breg function, thus serving as a promising candidate [107,108]. Clinical evidence further validates the increased saliva production in polysaccharide-treated SjD patients [109]. Moreover, alkaloids from *Dendrobium nobile* could protect against liver damage induced by carbon tetrachloride, which is associated with reduced mitochondrial oxidative stress [110]. In addition, growing evidence shows that gigantol effectively inhibits IL-6 signaling and oxidative stress pathways in murine nephritis [111,112], while polysaccharides from *Dendrobium* elicit therapeutic potential in treating inflammatory bowel disease (IBD) via the MAPK pathway [113]. The evidence demonstrates potent anti-inflammatory properties of *Dendrobium* that can be utilized to alleviate symptoms and inflammation associated with SjD.

### 4.4. Reishi Mushroom (Ganoderma lucidum)

*Ganoderma lucidum* is a medicinal fungus renowned for its immunomodulatory effects, particularly in regulating immune responses [114]. Studies suggest that *Ganoderma lucidum* boosts the activity of T and B cells, modulating gut microbiota and immune responses in inflammatory diseases and cancer development [115]. Moreover, *Ganoderma lucidum* can alleviate chronic inflammation by restraining effector T-cell counts, thus effectively alleviating the symptoms of sialadenitis and ameliorating glandular infiltration in a mouse model of SjD [116]. The immunomodulatory effects of *Ganoderma lucidum* make it a potential herbal candidate for treating SjD.

### 4.5. Goji Berry

Goji berries are widely utilized for their potent antioxidant and immunomodulatory properties [117,118,119]. Studies indicate that the polysaccharides and flavonoids in goji berries enhance the body’s antioxidant capacity, neutralize free radicals, and thereby mitigate immune dysregulation induced by oxidative stress [81]. Goji berries also have anti-inflammatory qualities that may ease the symptoms of SjD and various autoimmune disorders by reducing the release of pro-inflammatory cytokines [120]. Clinical data suggest that Goji berry extract positively impacts inflammatory diseases to ease symptoms, including fatigue, in patients [121].

### 4.6. Garlic

Garlic, a traditional herbal remedy, exhibits significant antioxidant, anti-inflammatory, and immunomodulatory properties. Garlic’s sulfur-containing compounds (e.g., allicin) are its key active ingredients, boosting the body’s immune response by modulating immune cell activity [122,123]. Recent findings show that garlic polysaccharides markedly increase the cytotoxic potential of immune cells and mitigate inflammatory responses by modulating the activities of diverse immune cell populations [124]. Several clinical trials indicate that garlic supplements significantly reduce oxidative stress levels in patients and, thus, alleviate inflammation [125], which may also be beneficial for patients with autoimmune diseases such as SjD.

### 4.7. Chrysanthemum

Chrysanthemum is a medicinal herb that possesses anti-inflammatory and antioxidant properties. Chrysanthemum contains compounds such as flavonoids, terpenes, and polysaccharides. These components, e.g., 6,8-C,C-diglucosylapigenin and eriodicyol-7-O-glucoside, show anti-inflammatory activity and can effectively inhibit the production of pro-inflammatory cytokines [126]. Additionally, the antioxidant components in chrysanthemum can scavenge free radicals in vitro and in vivo, thereby alleviating immune system damage caused by oxidative stress [127]. In diet-induced low-grade inflammation, the water extract of chrysanthemum can modulate the gut microbiota and increase SCFA levels [128], which may serve as a complementary diet in patients with inflammatory diseases, including SjD.

### 4.8. Lily

Accumulated pharmacological studies report that extracts and further compounds of lily possess a wide range of biological activities [129]. These properties suggest that lily could offer potential dietary approaches for alleviating symptoms associated with SjD. Lily exhibits its anti-inflammatory properties by modulating the expression of key inflammatory mediators, including IL-1β, TNF-α, inducible nitric oxide synthase (iNOS), and cyclooxygenase-2 (COX-2). By regulating these mediators, lily can help reduce inflammation and provide therapeutic benefits in various inflammatory conditions [130,131]. Studies have shown that lily extracts significantly inhibit the expression of iNOS, COX-2, IL-1β, IL-6, and TNF-α in RAW264.7 macrophage cells stimulated by LPS [132]. Additionally, in vivo studies have shown that lily extracts reduce the RNA expression levels of crucial inflammatory factors in the lung tissues of cigarette smoke-exposed mice. This indicates that lily extracts possess certain anti-inflammatory properties in the presence of cigarette smoke-induced lung inflammation [133]. Studies carried out by Chen et al. have indicated that a Lactobacillus brownii broth can reduce inflammation in tissues affected by chronic liver injury [134] by reducing the production of inflammatory cytokines, hence promoting the broth’s anti-inflammatory effect. This discovery shows the possible therapeutic effect of Lactobacillus brownii in the management of chronic liver inflammation. Therefore, moderate consumption of lily or its products, such as porridge, can have an adjuvant therapeutic effect in the management of patients with SjD.

The medicinal herbs discussed above—*Astragalus*, *Dendrobium*, *Ganoderma lucidum*, goji berry, garlic, chrysanthemum, and lily—exhibit a broad spectrum of immunomodulatory activities that converge on key pathogenic pathways reported in SjD. While each herb possesses distinct bioactive constituents and preferentially targets specific immune cell subsets, their mechanisms collectively span the regulation of T-helper cell polarization, enhancement of regulatory T- and B-cell function, modulation of gut microbiota, and attenuation of oxidative stress and pro-inflammatory cytokine production. These herbs often exert multi-target effects that align with the complex, heterogeneous nature of SjD, offering potential advantages over single-agent conventional therapies. However, clinical evidence remains limited, and most studies have been conducted using preclinical models. Future investigations should prioritize well-designed randomized controlled trials to validate efficacy, define optimal dosing regimens, and explore synergistic combinations that may harness the complementary mechanisms of different herbs. Thus, medicinal herbs may be rationally integrated into a comprehensive, personalized management strategy for SjD.

**Table 2:** Medicinal plants, active components, and immunomodulatory mechanisms.

Medicinal Plant	Bioactive Compounds	Mechanisms	References
*Reishi Mushroom* (*Ganoderma lucidum*)	*Polysaccharides* (*Ganoderma polysaccharides*)	Enhances the activity of T cells and B cells, modulates immune responses, and alleviates chronic inflammatory responses.	[79]
*Dendrobium* (*Dendrobium officinale*)	Polysaccharides (*Dendrobium polysaccharides*) and Alkaloids	Reduces levels of inflammatory cytokines, inhibits the expression of retinal VEGF, protects the liver, and alleviates dry eye and systemic inflammatory states in SjD.	[80]
*Goji Berry* (*Lycium barbarum*)	*Polysaccharides* (*Lycium polysaccharides*)	Enhances the body’s antioxidant capacity, scavenges free radicals, alleviates immune dysregulation caused by oxidative stress, and alleviates symptoms of SjD and other autoimmune diseases.	[81]
*Garlic* (*Allium sativum*)	Sulfur-containing compounds	Regulates the activity of immune cells, enhances the body’s immune response, significantly enhances the cytotoxicity of the immune system, and alleviates inflammatory responses.	[86]
*Chrysanthemum* (*Chrysanthemum morifolium*)	Flavonoids, terpenes, and polysaccharides	Possesses significant anti-inflammatory activity, effectively inhibits the production of pro-inflammatory cytokines, scavenges free radicals, and alleviates damage to the immune system caused by oxidative stress.	[87]
*Astragalus* (*Astragalus membranaceus*)	*Polysaccharides* (*Astragalus polysaccharides*)	Promotes T-cell proliferation, enhances the signaling of T-cell receptors (TCRs) and co-stimulatory molecules, activates PKC and tyrosine kinases, promotes the activation of NF-κB and nuclear factor of activated T-cells, and enhances the expression of cytokines.	[92]
*Lily* (*Lilium brownii*)	Polyphenols	Regulates the expression of various inflammatory mediators, significantly inhibits the expression of inflammatory factors, and alleviates inflammatory responses in SjD.	[130,132]

## 5. Conclusions

The intricate interplay between diet, immunity, and autoimmunity offers a challenge in its complexity and an opportunity to intervene at the roots of immune dysregulation. Nutritional and herbal strategies hold considerable promise as complementary approaches in the management of SjD. By targeting key immunological checkpoints—ranging from T- and B-cell subset differentiation to metabolic reprogramming and gut microbiota modulation—these interventions offer a multifaceted means of restoring immune homeostasis. Unlike conventional immunosuppressants, which often carry significant toxicity and lack sustained efficacy, dietary components and medicinal herbs can engage diverse bioactive pathways simultaneously, aligning well with the complex pathophysiology of SjD. Nevertheless, translating these promising findings into clinical practice will require more than mechanistic insights. Future efforts must prioritize rigorous, well-controlled clinical trials to establish efficacy, safety, and patient-specific response profiles. Moreover, integrating modern analytical technologies—such as metabolomics, single-cell immunophenotyping, and gut microbiome sequencing—will be essential to decoding the molecular interplay between diet, herbal bioactives, and host immunity. As we stand at the intersection of traditional knowledge and precision medicine, the path forward lies not in replacing conventional therapy but in thoughtfully weaving nutritional and herbal interventions into a holistic, individualized treatment framework. In doing so, we may move beyond symptomatic management toward a more sustainable, immune-resetting approach for Sjögren’s disease and other autoimmune conditions.

## Data Availability

The original contributions presented in this study are included in the article. Further inquiries can be directed to the corresponding author.
